# Effect of Ce-doped bioactive glass/collagen/chitosan nanocomposite scaffolds on the cell morphology and proliferation of rabbit’s bone marrow mesenchymal stem cells-derived osteogenic cells

**DOI:** 10.1186/s43141-022-00302-x

**Published:** 2022-02-21

**Authors:** Hanan F. Hammouda, Mohammad M. Farag, Mervat M. F. El Deftar, M. Abdel-Gabbar, Basant M. Mohamed

**Affiliations:** 1 The Healthy Chemistry Department, Center Health Laboratory, Ministry of Health, Cairo, Egypt; 2 Glass Research Department, National Research Centre, 33 El Bohouth Str., Dokki, Giza, 12622 Egypt; 3 Department Pathology, Tissue Culture and Cytogenesis Unit, National Cancer Institute, Cairo University, Cairo, Egypt; 4 Departmentof Chemistry, Faculty of Science, Beni-Suef University, Beni-Suef, Egypt

**Keywords:** Nanobioactive glass, Cerium, Chitosan, Collagen, Scaffold, Mesenchymal stem cells, Osteogensis

## Abstract

**Background:**

Cerium-containing materials have wide applications in the biomedical field, because of the mimetic catalytic activities of cerium. The study aims to deeply estimate the biocompatibility of different scaffolds based on Ce-doped nanobioactive glass, collagen, and chitosan using the first passage of rabbit bone marrow mesenchymal stem cells (BM-MSCs) directed to osteogenic lineage by direct and indirect approach. One percentage of glass filler was used (30 wt. %) in the scaffold, while the percentage of CeO_2_ in the glass was ranged from 0 to 10 mol. %. Cytotoxicity was evaluated by monitoring of cell morphological changes and reduction in cell proliferation activity of BMMSCs maintained under osteogenic condition using proliferation assays, MTT assay for the direct contact of cells/scaffolds twice in a week, trypan blue and hemocytometer cell counting for indirect contact of cells/scaffolds extracts at day 7. Cell behaviors growth, morphology characteristics were monitored daily under a microscope and cell counting were conducted after 1 week of the incubation of the cells with the extracts of the four composite scaffolds in the osteogenic medium at the end of the week.

**Results:**

Showed that at 24 h after direct contact with composite scaffold, all scaffolds showed proliferation of cells > 50% and increased in cell density on day 7. The scaffold of the highest percentage of CeO_2_ in bioactive glass nanoparticles (sample CL/CH/C10) showed the lowest inhibition of cell proliferation (< 25%) at day 7. Moreover, the indirect cell viability test showed that all extracts from the four composite scaffolds did not demonstrate a toxic effect on the cells (inhibition value < 25%).

**Conclusion:**

The addition of CeO_2_ to the glass composition improved the biocompatibility of the composite scaffold for the proliferation of rabbi**t** bone marrow mesenchymal stem cells directed to osteogenic lineage.

**Supplementary Information:**

The online version contains supplementary material available at 10.1186/s43141-022-00302-x.

## Background

Fabricated scaffold acting as a temporary substitute for bone defects is still under investigation, and so far the scientists have not been able to get an ideal scaffold that overcomes all problems associated with the traditional methods of bone surgery by bone-graft substitutes like morbidity of the donor site and limitation in the amount of bone available and surgical cost for the harvesting procedure also, are highly associated to the risk of rejection, infection, contamination, pain at the harvest site, bleeding hematoma, and disease transfer [1–3], lack of osteogenicity and most of them lack osteoconductivity as implant materials of low biocompatibility such as copper, silver, and bone cement shows little or no osteoconduction, while other carry hazards of viral transmission and other implants like metals would release harmful ions and increase the risk of cancer [4, 5]. The ideal scaffold should be biocompatible, i.e., non-immunogenic and non-toxic to be in contact with the living system without producing an adverse effect that might reduce healing or cause rejection by the body [6].

Recently, the intensity of research on hybrid or composite scaffolds is rapidly increasing, especially in the field of tissue engineering. These composite materials have been developed to combine different material properties to overcome some drawbacks related to some materials and obstacles to their use in important applications.

Based on the literature, composite scaffolds used in tissue engineering applications are either synthetic or naturally derived polymers. Natural polymers attracted the attention of researchers toward the development of suitable biocompatible composite scaffolds for use in bone tissue regeneration. This is due to natural polymers being more available, superabundant, and similar to the extracellular matrix components. Collagen is the major constituent of natural bone and has dual properties in the bioactivity as well as in the biomimetic property. It offers many binding sites for cell attachment, increasing the growth and proliferation of cells, as reported in several studies [7, 8]. However, the low mechanical strength and osteoinductivity of collagen limit its wider applications in the bone regeneration field. By incorporating different biomaterial’s, the properties such as porosity, structural stability, osteoinductivity, and osteogenicity of collagen matrixes can be largely improved [9].

Chitosan is another type of natural polymer derived from renewable marine resources and industries from the chitin of crustaceans and fungal mycelia. It is a semi-crystalline polysaccharide polymer. It is composed of N-acetyl d-glucosamine and d-glucosamine units [10, 11]. Bioactive compounds as well as bioresorbable materials, which can mimic the natural function of bone and activate in vivo mechanisms for bone regeneration. They can be easily functionalized to enhance bone mineralization [12]. Moreover, the favorable properties, such as biodegradability, mucoadhesion, hemostatic activity, its antibacterial and antifungal activities, cell compatibility, and limited immunogenicity [10, 11], directed this polymer to be widely used in different biomedical applications. However, chitosan-based scaffolds have limitations in terms of mechanical strength and osteoconductivity hinders its application in bone tissue engineering. To overcome these limitations, chitosan has been blended with a variety of materials that include (natural and synthetic) polymer, ceramics, and other additives [13].

The combination of two or more polymers allows to develop new biomaterials that exhibit combinations of properties that could not be obtained from individual polymers [14]. Therefore, there have been numerous studies that used chitosan and collagen to fabricate scaffolds for skin regeneration [15–17] and vascular regeneration [18–20].

For bone regeneration, polymer–ceramic composites are considered an advanced class of biomaterial’s that are more optimal for bone scaffolding applications due to the biodegradable polymers that are usually not bioactive; therefore, incorporation of bioactive materials into these biodegradable polymers combines between the bioactivity property, which comes from the bioactive materials and gains the flexibility characteristic which comes from the polymer [21, 22]. The bioactive glasses are amorphous materials (usually based on silica) that are biocompatible, bioactive, osteoconductive, and even osteoproductive, that comes from their unique ability to convert to hydroxyapatite in vivo, and their capability to bond with the bone and soft tissues [23–25] which recommended them as very suitable filler for biopolymers matrices [26, 27]. Moreover, bioactive glasses are characterized by a possibility to incorporate therapeutic ions (e.g., Li, Ag, Cu, and Ce). Cerium is one of the potential therapeutic ions added to bioactive glasses. It can protect the cells from damage created by the reactive oxygen species (ROS) which resulted from normal oxygen metabolism [28]. That is due to their ability to switch the oxidation states between Ce^4+^ and Ce^3+^ [29]. This nominated their compounds, such as nanoceria, to be used in the treatment of several diseases and disorders, including cardiovascular disease, Parkinson’s and Alzheimer’s disease, and even tumor development [30]. Thus, cerium has been incorporated in numerous bioactive glasses to improve their functionality for the treatment of several diseases [31–41].

In order to improve and accelerate the bone healing process, the involvement of mesenchymal stem cells (MSCs) with bone composite scaffold is critical, MSCs are multipotent stromal stem cells that can be harvested from many different sources and differentiated into different lineages such as adipogenic, chondrogenic, and osteogenic; however, aging, senescence, and oxidative stress reduce their ex vivo expansion, which is critical for their clinical applications. Therefore, there is a great need to identify methods to manipulate MSCs to reduce ROS in both the MSCs themselves during their culture expansion production phase and in the injured tissue microenvironment to promote MSC engraftment and enhance tissue repair. It has been reported that antioxidants stimulate MSC proliferation [42]. Concerning the osteogenesis process, numerous studies point to ROS inhibiting osteogenic differentiation [43]. In MSCs, excess ROS or exogenous addition of H_2_O_2_ can weaken self-renewal, differentiation capacity, and proliferation [44–46] especially that MSCs are rare cells; they constitute only 0.001 to 0.01% of the bone marrow population. Since regeneration of large tissues requires around 10^7^ to 10^8^ MSCs [47], there exists a need for MSCs to be expanded prior to tissue regeneration. Several passages in vitro decrease the life span of cells, as their longevity and functions are affected by oxidative stress; hence, antioxidants stimulate MSC proliferation [42]. The idea is that ROS and oxidative stress must decrease to allow for osteogenic differentiation to implement.

Accordingly, incorporation of Ce-containing bioactive glasses in biodegradable polymer scaffolds increases the impact and value of these scaffolds for tissue engineering applications, and there have been several studies that used bioactive glasses doped with cerium as bioactive fillers in polymer scaffolds [48–51]. Biocompatibility testing became an important step toward animal testing and finally clinical trials that will determine the biocompatibility of the material in a given application, and thus medical devices, like implants or any other stimulating delivery devices, such as proteins, genes, and drugs [52]. The biocompatibility testing contains numerous in vitro tests that are used following ISO (10993) or other standards as defined by IUPAC [53] qualitatively by the *direct contact and* quantitatively by MTT assay.

This work aims to fabricate suitable biocompatible composite scaffolds based on collagen and chitosan polymer blend used as a polymer matrix for nanobioactive glass doped with different ratios of CeO_2_ next to study the morphological and microstructural characterization of those scaffolds to know whether they will facilitate the proliferation of BMMSCs directed to osteogenic lineage to be used for basic research studies and future tissue engineering purposes. According to our knowledge, there have not much data about the biocompatibility of composite scaffolds based on collagen and chitosan polymer blend used as a polymer matrix for nanobioactive glass doped with different ratios of CeO_2_. In vitro biocompatibility test was performed by evaluating cell morphology and measuring cell viability by the direct contact of the BMMSCs directed to osteogenic lineage around or in the vicinity of the scaffold surfaces and by the indirect test using the fluid extracts of these scaffolds for the proliferation of cells.

## Methods

### Preparation of nanobioactive glasses (NBGs) and scaffolds

Different nanobioactive glasses were synthesized based on (80-x) SiO_2_-15CaO-5P_2_O_5_-xCe_2_O, in mole % (*x* = 0, 5, and 10 mole %); accordingly, the glass was encoded as BG-C0, BG-C5, and BG-C10, respectively (Table 1). The glasses were prepared by a quick alkali-mediated sol-gel method [54]. Briefly, TEOS was added to EtOH solution and then 2M HNO_3_ was added, the molar ratio of TEOS: H2O: EtOH was 1:8:10. Subsequently, TEP, Ca(NO_3_)_2_·4H_2_O, and ammonium cerium (IV) nitrate were added, respectively, after complete hydrolysis of TEOS, with 20 min time interval, and the solution left to stir for 3 h. The sol was subjected to quick gelling using a concentrated ammonia solution. The resulted gel was dried at 60 °C for 2 days and ultimately it was transformed to glass by calcination at 600 °C in the air for 30 min. The obtained glass particles were used subsequently in preparation of composite scaffolds.

**Table 1:** Glass composition in mole %

	SiO_2_	CaO	P_2_O_5_	CeO_2_
C0	80	15	5	0
C5	75	15	5	5
C10	70	15	5	10

The scaffolds used in this study were prepared based on collagen and chitosan, with 1:1 volume ratio, as a polymer matrix, and the glass was added to the scaffold with 30 wt.%. Typically, 2% (w/v) of chitosan was dissolved in 1 % (v/v) acetic acid. An equal volume of chitosan solution and as-prepared collagen solution was mixed well, and the glass particles were added to this mixture and stirred for 3 h and put in the ultrasonic bath for 30 min to assure dispersion of glass particles in the polymer matrix. And then, the mixture was frozen at –20 °C for 2 days and lyophilized thereafter at −50 °C for 2 days to obtain the final scaffold [55]. The collagen/chitosan, collagen/chitosan/C0, collagen/chitosan/C5 and collagen/chitosan/C10 scaffolds encoded as CL/CH, CL/CH/C0, CL/CH/C5, and CL/CH/C10 samples, respectively.

### Characterization of nanobioactive glass and derived scaffolds

Bioactive glass nanoparticles were characterized by DTA (differential thermal analysis) by using Setaram, in the temperature range 25‑800 °C with a rate of 10 °C.min^−1^ to determine the thermal behavior of the dry gels. A transmission electron microscope (TEM, model; JEM2010, Japan) working at 100 kV, was used to investigate the morphology and particle size. Furthermore, Fourier transform infrared (FTIR) technique was used to detect the characteristic vibration modes in the wavenumber range of 4000–400 cm^−1^ by using JASCO FT/IR-4600.

The morphological and microstructural characterizations of the scaffolds were examined by SEM/EDX (model, HITATCHI Su800). Scanning electron microscopy (SEM) on the gold-coated specimen was used to examine the morphological and textural features of the sample, using an accelerating voltage of 15 kV. Before scanning, discs were sectioned with a very sharp scalpel in order to expose their internal (cross-sectional) microstructure. Sectioning was necessary because the surfaces of the external disc exhibited much smoother architecture than their internal parts.

### In vitro biocompatibility test

#### Cell isolation

**Fig. 1:**
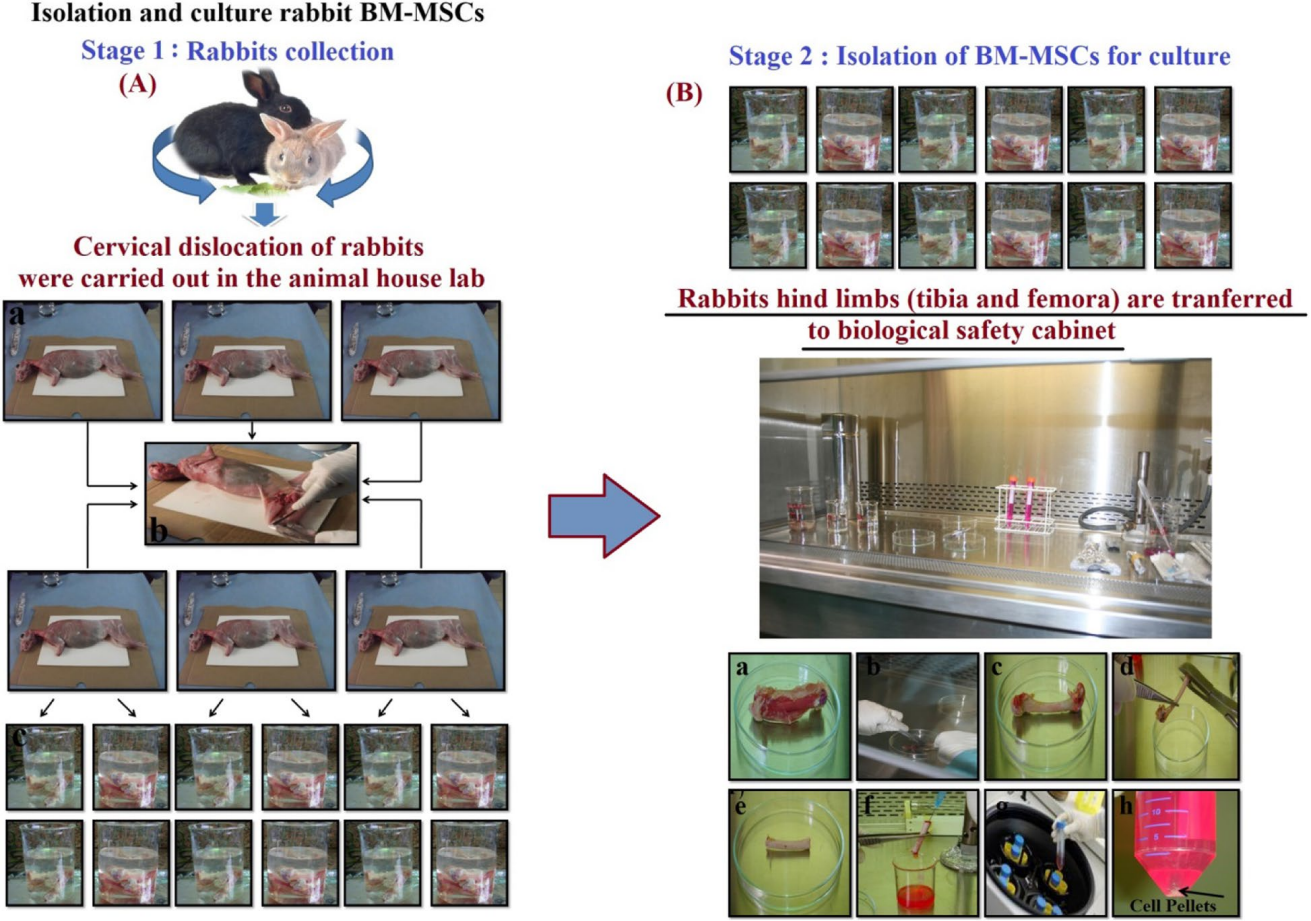
Steps of BM-MSCs isolation, *stage 1:* Steps for dissection of Rabbit’s hind limbs (tibia and femora): (**a**) Animal is fixed on carton board using metal pins (arms and legs) and swabbed with 80% isopropanol then (**b**) the pelt was clipped and peeled back to expose the hind limbs on two sides. (**c**) Bones are immersed in alcohol for 15 min and then transferred to the biological safety cabinet. *Stage 2:* The steps of bone marrow isolation from dissected hind limbs of rabbits inside the biological safety cabinet: (**a**‑**c**) Remaining muscle tissue was removed. (**d**, **e**) The two femur and tibia heads were cut with a bone cutter. (**f**) A medium-filled syringe was used to flush the marrow out of the femur and tibia bones. (**g**) The cell suspension was transferred to15 ml centrifugation tube and centrifugation was done at 1000 rpm for 5 min. (**h**) Cell pellets (black arrow) were re-suspended in 1 ml complete expansion medium

The osteoblast-like cells were obtained from bone marrow mesenchymal stem cells (BM-MSCs) isolated from the femurs and tibia of six rabbits with an (average age: 6‑8 weeks and average weight: 0.75‑1 kg). The rabbits were sacrificed without complication to animals by cervical dislocation in the animal house lab using the guidelines approved by the Medical Research Ethics Committee on the Use of Animal Subjects at National Cancer Institute, Cairo, and approved by the Ethics Research Committee of the Faculty of Science, Beni-Suef University, Beni-Suef, Egypt (Ethics approval number: 021-194). Figure 1 represents the steps of isolation of BM-MSCs from the rabbit’s hind limbs (tibia and femora). The isolation protocol was performed according to protocols described previously [56–58] on two stages, the first steps (Fig. 1A (a, c)) for dissection of rabbit’s hind limbs (tibia and femora) in animal house lab, the animal is a fixed carton board using metal pins (arms and legs) and swabbed with 80% isopropanol and then the pelt was clipped and peeled back to expose the hind limbs on two sides after that bones are immersed in alcohol for 15 min after that transferred to the biological safety cabinet. The second step for bone marrow isolation from dissected hind limbs of rabbits inside the biological safety cabinet (Fig. 1B (a‑h)). Remaining muscle tissue was removed. The two femur and tibia heads were cut with a bone cutter. A medium-filled syringe was used to flush the marrow out of the femur and tibia bones and then the cell suspension was transferred to a 15 ml centrifugation tube and centrifugation was done at 1000 rpm for 5 min and finally cell pellets were re-suspended in 1 ml complete expansion medium.

Cell morphology study of the four composite scaffolds (direct contact) and their extracts (indirect contact) was examined using inverted phase contrast microscopes; a qualitative method was used to test the biocompatibility of the material by placing it directly with cultured cells.

Cells were collected and cultured in expansion medium low-glucose DMEM/Ham’s F12 (Biochrome, AG) mixture (1:1) supplemented with 10% fetal bovine serum (Biochrome AG), 10% basic-Fibroblast growth factor (Sigma), 1% l-glutamax (Invitrogen, 2 mm), 1% penicillin-streptomycin (100 U/ml-100 μg/ml, Lonza), and 0.25 μg fungizone (Lonza) and then incubated in a humidified atmosphere of 5% CO_2_/95% air at 37 °C. The medium was completely exchanged every 3 days till reached to confluence 80% to 90%. Cells were trypsinized with 0.25% trypsin (Lonza) and collected by centrifuge and then counted with a hemocytometer and re-plated again as the first passage at a density 5 × 10 ^3^cells per/cm^2^ in 24-well culture plates for further expansion and osteogenesis. Cells were maintained in osteogenic medium (the same expansion medium described above plus 50 μm l-ascorbic acid 2-phosphate, 100 nm dexamethasone, and10 mm β-glycerophosphate (Applichem)) [59].

The reactivity of the four composite scaffolds (CL/CH, CL/CH/C0, CL/CH/C5, and CL/CH/C10) was indicated by studying cell morphology in the vicinity of scaffold surfaces in culture wells and observing malformation, degeneration, and lysis of cells around the test material in comparison to non-toxic negative control material.

The direct contact was done by seeding the isolated sub-cultured BM-MSCs cells at a density 5 × 10 ^3^cells per/cm2 in 24-well culture plates, first for 1 day in osteogenic medium and then leaving the cells to settle first by plastic adherence property (specific property for mesenchymal stem cells) after that, we put the gamma rays sterilized composite scaffolds which maintained in the osteogenic cultured medium at least 12 h before incubation (CL/CH, CL/CH/C0, CL/CH/C5, and CL/CH/C10) respectively at the corners of well plates not directly on the cells to avoid cells disturbance. Also, we do independent repeatability for each scaffold to be easy to do good analysis. All composite scaffolds were incubated in 24-well culture plates with sub-cultured BM-MSCs for 1 week in a humidified atmosphere of 5% CO_2_/95% air at 37 °C for 7 days.

The morphology, cell spread, and cell growth were microscopically evaluated daily till day 7. The scaffolds planned for phase-contrast microscopic examination were originally prepared in very thin sections to give adequate translucency that would allow the light to pass through and then were cut in pieces equal in size 100 mg/ml and shape using sterile scissors and scalpels.

The cell-seeded scaffolds were directly examined without fixation after 24 h of culture while they were still in their culture plate. Also, groups of cell/scaffolds were examined by fixation and staining with Giemsa stain after 3 days of culture and day 7. Many pictures of cells were taken at different time points for studying morphological features and the capacity to adhere to plastic plates.

Another hand, a group of cells in the 24 well plates (in triplicate) was left to incubate in the various extract composite scaffolds prepared in the osteogenic medium as described by Zhou and Chen [60]. All the same pieces were immersed in the extracting media 100 mg/ml preparation of the different extracts. The extract media were the same culture osteogenic medium as described above. The extraction process was carried out in a water bath at 37 °C in separate four 15 ml falcon tubes and then was shaken at a speed of 60–65 rev/min for 2 days. The extracts were passed through a 0.22-μm filter and then were stored at –20 °C till used for at least 1 week.

The four composite scaffold samples and their extract were maintained under the same culture conditions alone without cells besides the tissue culture plastic polystyrene with culture media with cells was used as a non-toxic negative control material and the same four composite scaffolds and their extracts were supplemented with polyvinyl chloride (concentration of 20 and 100 mg/ml) in culture medium was used as a positive toxic control material. Both seeded and unseeded were examined daily.

#### MTT proliferation assay

The biocompatibility of the four composite scaffolds was assessed colorimetrically by MTT assay [61]. After incubation, the scaffold pieces were removed. The cells were rinsed with PBS to remove non-adhering cells, followed by incubation in 50 μl MTT reagent (5 mg/ml) in PBS for 4 h. Formazan crystals formed were dissolved by adding 500 μl of DMSO solution to lyse cells and release formazan. The solution (150 μl) from each sample was transferred to 96 well-plates, and optical density (O.D) was measured in a BMG LABTECH®- FLUOstar Omega microplate reader (Ortenberg, Germany) at an absorbance of 490 nm [62]. The tissue culture plastic polystyrene was used as non-toxic negative control material and polyvinylchloride (concentration of 20 and 100 mg/ml) was used as the positive toxic control material. Culture plates were incubated in a humidified atmosphere of 5% CO_2_/95% air at 37 °C and the MTT assay was performed two times at hour 24 and day 7 in triplicate by measuring optical density then evaluating cell growth % and cell inhibition % as follows.

$$\%\kern0.5em \mathrm{Cell}\kern0.5em \mathrm{growth}\kern0.5em \left(\mathrm{cell}\kern0.5em \mathrm{viability}\right)=\frac{\mathrm{Absorbance}\kern0.5em \left(\mathrm{O}.\mathrm{D}\right)\kern0.5em \mathrm{of}\kern0.5em \mathrm{sample}}{\mathrm{Absorbance}\kern0.5em \left(\mathrm{O}.\mathrm{D}\right)\kern0.5em \mathrm{of}\kern0.5em \mathrm{negative}\kern0.5em \mathrm{control}}\times 100$$

$$\%\kern0.5em \mathrm{Cell}\kern0.5em \mathrm{inhibition}=100\hbox{-} \%\kern0.5em \mathrm{cell}\kern0.5em \mathrm{growth}$$

Another hand (indirect contact test), we evaluated the reduction in cell proliferation activity of the four composite extracts by the trypan blue exclusion test of cell viability in Neubauer hemacytometer at day 7 in triplicate [63]. The old medium in well culture plates of sample extracts was discarded, and the adherent cells washed with 1 ml of Dulbecco’s phosphate-buffered saline without calcium and magnesium (DPBS-A) ions to remove traces of serum presented in the medium. The cells were detached from well plates using 0.25% trypsin with 0.1 % EDTA thereafter, and then transferred into 15 ml centrifuge tubes and centrifuged at 104 rpm for 5 min. Briefly, cells were re-suspended in PBS containing trypan blue by mixing 1 part of 0.4% trypan blue and 1 part cell suspension (dilution of cells) and allow the mixture to incubate ∼3 min at room temperature and then were examined in counting chamber of hemacytometer by inverted phase-contrast microscope to determine the percentage of cells that have clear cytoplasm, non-stained (viable cells) versus cells that have blue cytoplasm stained (nonviable cells). The percentage of viable cells was measured as follows:

$$\mathrm{Viable}\kern0.5em \mathrm{cells}\kern0.5em \%=\frac{\mathrm{Total}\kern0.5em \mathrm{number}\kern0.5em \mathrm{of}\kern0.5em \mathrm{viable}\kern0.5em \mathrm{cells}\kern0.5em \mathrm{per}\kern0.5em \mathrm{ml}\kern0.5em \mathrm{of}\kern0.5em \mathrm{aliquot}}{\mathrm{Total}\kern0.5em \mathrm{number}\kern0.5em \mathrm{of}\kern0.5em \mathrm{cells}\kern0.5em \mathrm{per}\kern0.5em \mathrm{ml}\kern0.5em \mathrm{of}\kern0.5em \mathrm{aliquot}}\times 100$$

### Statistical analyses

All experimental data stated in this work were expressed as the average ± standard deviation (SD) for *n* = 3 and were analyzed using standard analysis of the one-way ANOVA test. The level of significance (*p* value) is set at < 0.05.

## Results

### DTA

**Fig. 2:**
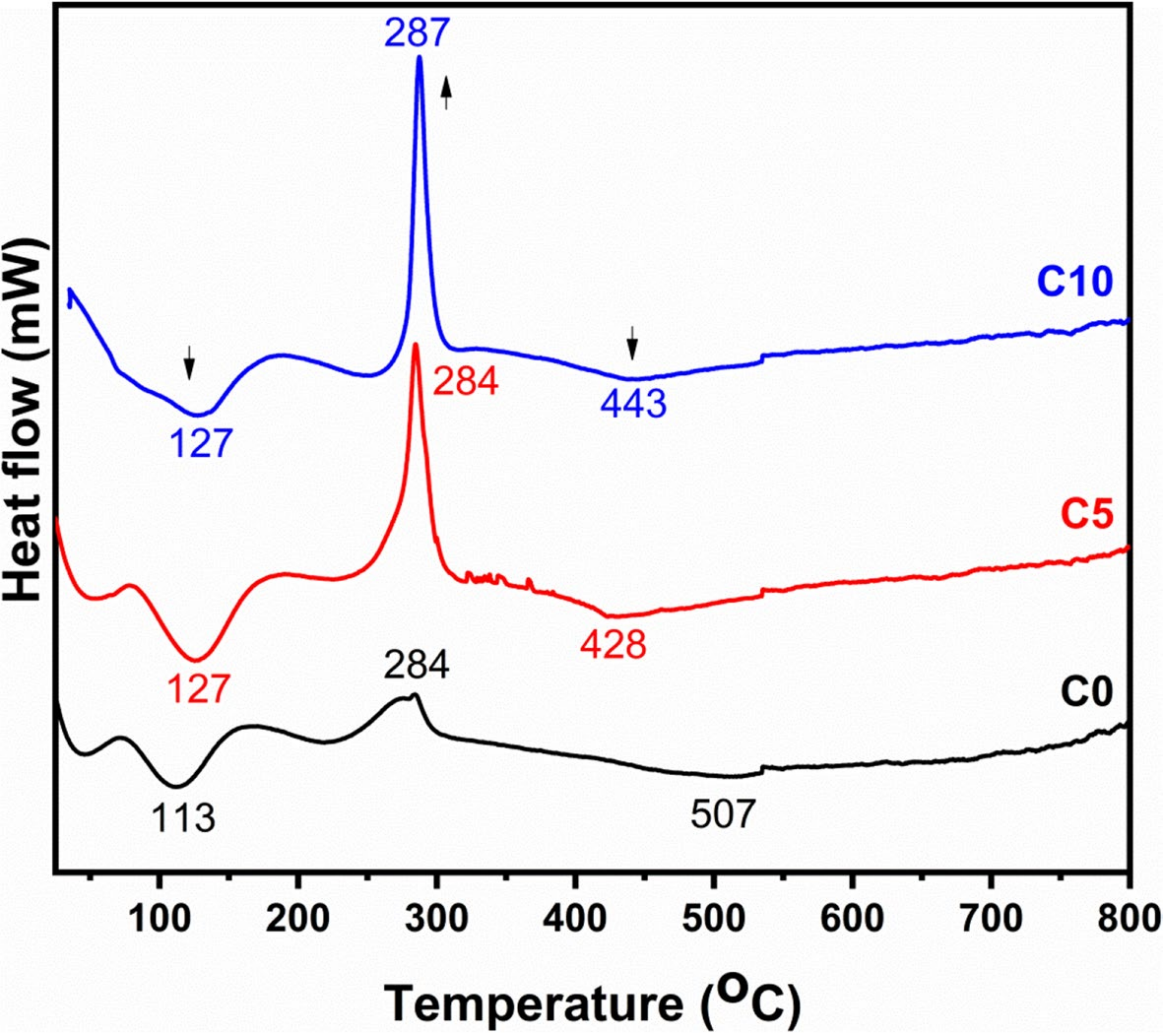
The differential thermal analysis (DTA) of dry gels, C0, C5, and C10

The differential thermal analysis (DTA) of dry gels, C0, C5, and C10 are shown in Fig. 2. The first endothermic peaks at 113 °C, 127 °C, and 127 °C for C0, C5, and C10 (see Table 1), respectively, were assigned to the elimination of adsorbed water from the dry gels [64]. The exothermic peaks observed at 284 °C, 284 °C, and 287 °C for C0, C5, and C10, respectively, were attributed to the removal of organic species of the starting materials (i.e., alkoxyl groups). Moreover, the second endothermic peaks detected at 507 °C, 428 °C, and 443 °C for C0, C5, and C10, respectively, were attributed to the further removal of organics in addition to the loss of residual nitrates of the precursors, as well as the glass transition temperatures (Tg) [65]. The results showed that the residuals were totally removed before 510 °C for all samples. Accordingly, the temperature of 600 °C was selected for the calcination of the as-prepared dry gels to obtain the glass powders. It can be noticed from these results that the addition of CeO_2_ to the glass was decreased the Tg, this can be explained by disrupting the effect of such oxide on the glass network, and hence the generation of more number of non-binding oxygen in the network [41]. These results were confirmed by FTIR analysis.

### TEM

**Fig. 3:**
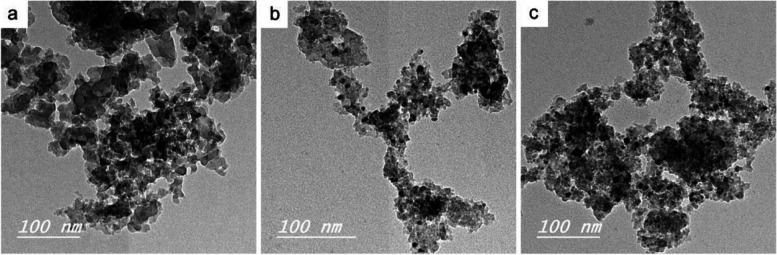
TEM micrographs of C0, C5, and C10 glasses (**a**, **b**, and **c**, respectively)

The morphology and particle size of different glass samples were examined by TEM technique (Fig. 3) shows TEM micrographs of C0, C5, and C10, respectively. It can be noted from the figure that the particle sizes of all samples were in the nano-scale (< 100 nm) with spherical shapes. Furthermore, and the Ce-substituted glasses (C5 and C10 samples) were obviously smaller than that of Ce-free glass (C0). The particle size ranged from 30 to 80 nm for C0 glass, while it was ranged from 10 to 50 nm for C5 and C10 glasses. Therefore, the substitution of Ca with Ce in the glass composition caused a decrease in the particle size.

### FTIR

**Fig. 4:**
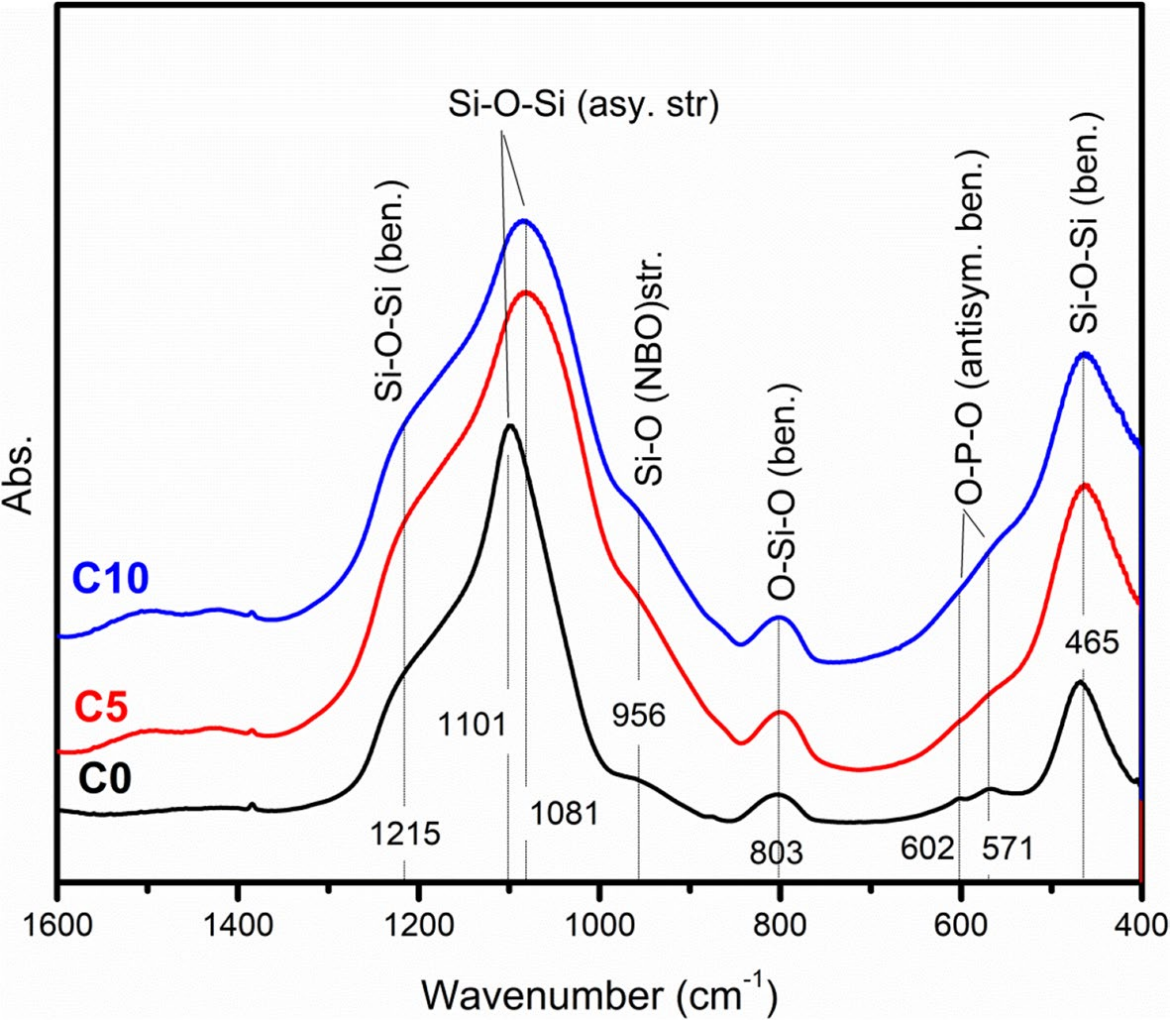
FTIR of the vibration modes of C0, C5, and C10 glasses

The vibration modes of different units in the glass network of different glass samples were investigated by FTIR as shown in Fig. 4. It can be noted from the figure presence of a peak at 465 cm^−1^for all samples, which was attributed to Si-O-Si bending vibration modes [33]. Very weak doublets at 571 and 602 cm^−1^ were observed clearer in C0 glass than that of the other two glasses which were assigned to the antisymmetric bending mode of O-P-O in the phosphate groups (PO_3_^−4^). Moreover, O-Si-O bending mode of orthosilicate SiO_4_^4−^ was located at 803 cm^−1^ [66]. The shoulder detected at 956 cm^−1^ was assigned to Si-O-NBO stretching [67]. It was stronger in C5 and C10 than in C0, which indicated that the addition of CeO_2_ at expense of CaO disrupted the silicate glass network and increased NBOs throughout the network [68]. The presence of a large number of NBOs increases the reactivity of the glass surface in the biological fluid and hence stimulates apatite formation with the surrounding tissues [69]. The Si-O-Si asymmetric stretching vibration mode was noted at 1101 cm^−1^ for C0 sample and 1081 cm^−1^ for C5 and C10 glasses [33]. Accordingly, CeO_2_ shifted this band toward the lower value, because it weakened the glass network. Finally, the broad shoulder noticed at about 1215 cm^−1^ was assigned to Si-O-Si bending mode [70].

### Composite scaffolds morphology

**Fig. 5:**
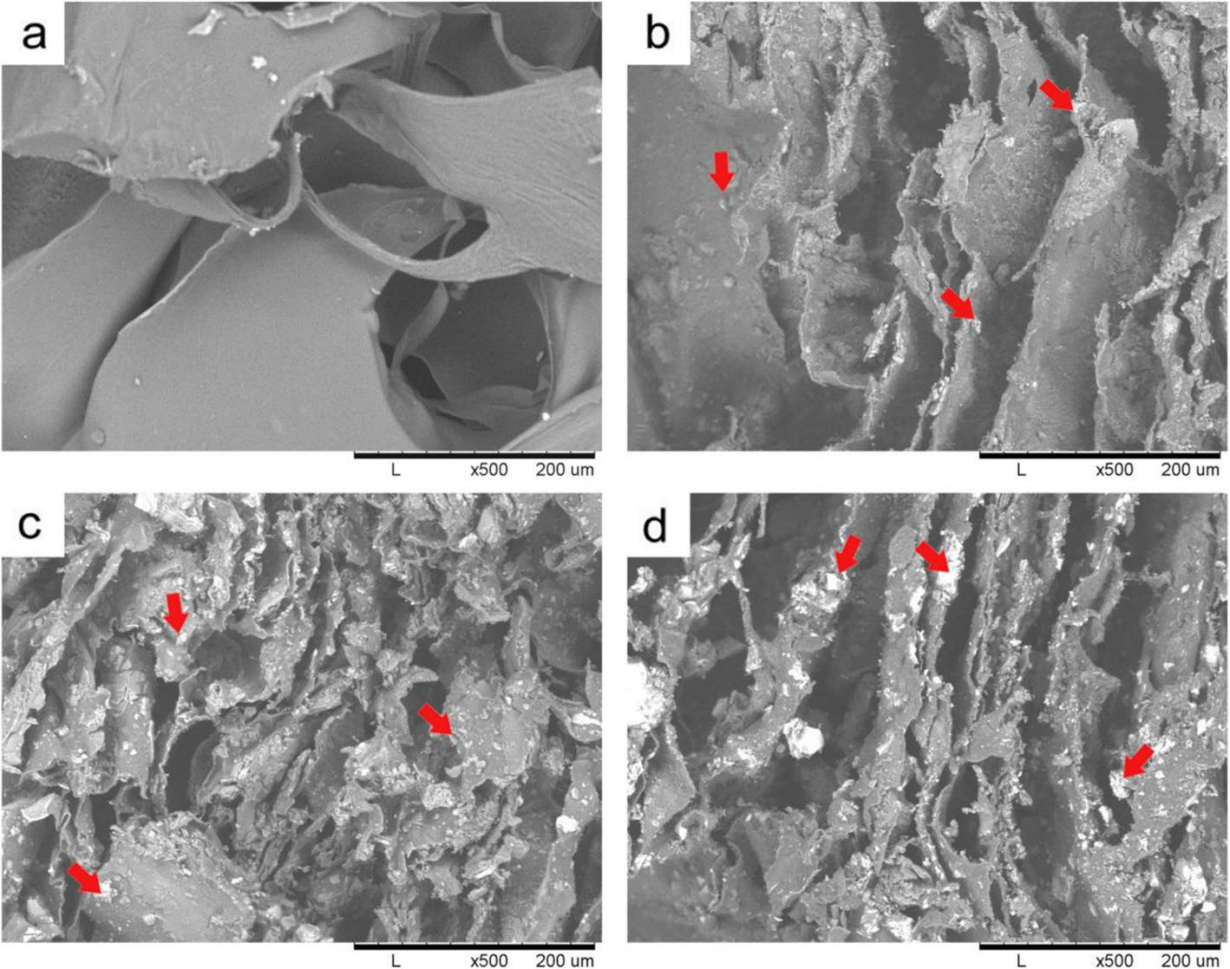
SEM micrographs of the composite scaffolds, CL/CH, CL/CH/C0, CL/CH/C5, and CL/CH/C10 samples (**a**, **b**, **c**, and **d**, respectively). Red arrows pointed to the glass particles distributed in the polymer matrix

Different composite scaffolds based on collagen, chitosan, and/or nano-bioactive glass (NBG) were prepared by the thermal-induced phase separation method, and the cross-section of these scaffolds was examined by SEM technique. Figure 5 shows SEM micrographs of CL/CH, CL/CH/C0, CL/CH/C5, and CL/CH/C10 scaffolds. From the figure, it can be noticed that all scaffolds were characterized by macroporous and interconnected porous structures. On the other hand, the neat collagen/chitosan scaffold (sample CL/CH) possessed a circular and relatively large pore size (50–300 μm), while the scaffolds that contained bioactive glass fillers were characterized by elongated and a relatively small pore size. Moreover, the figure showed a well-distribution of glass particles in the polymer matrix. Accordingly, the addition of glass nanoparticles to the polymer was showing a considerable effect on the pore morphology and the pore size of the final scaffolds.

### Isolation and in vitro expansion of MSCs

**Fig. 6:**
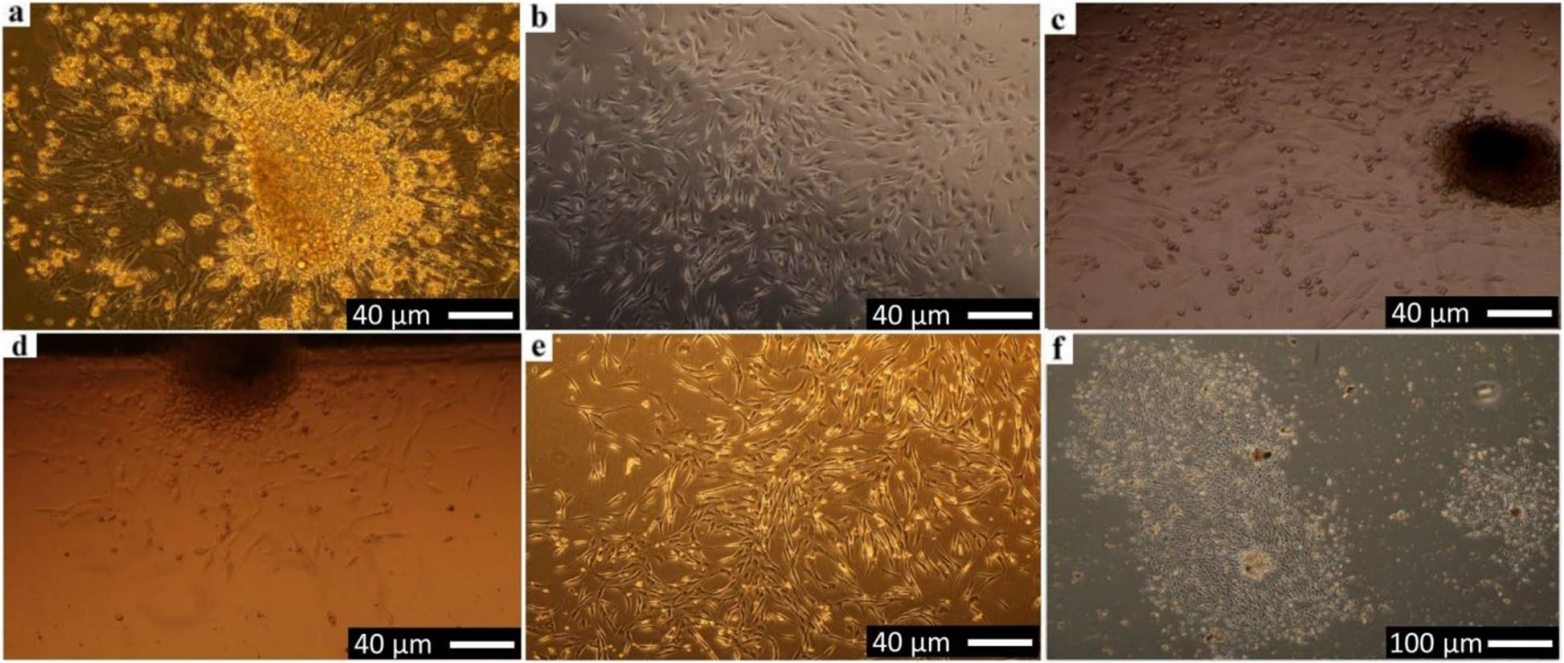
Inverted phase-contrast micrographs show the morphology of primary cultured Rabbit BM-MSCs after removal of non-adherent cells in expansion medium (**a**-**d**) at days 3-4, (**e**) confluence (80-90%) at days 5-7, (**f**) Nodular confluence (colonies varied from 3-7 per flask T25 cm^2^)

Cells were obtained by bone marrow flashing and seeded at a high primary seeding density of 1 × 10^5^ BM mononuclear cells and day 2; by inverted phase-contrast microscope, elongated cells appeared and were selected by adherence to plastic, whereas floating cells are residual red blood cells and unattached mononuclear cells were maintained in expansion medium. On day 3, following removal of non-adherent hematopoietic stem cell populations by changing medium for the first time, colonies were formed of rounded, yellowish cells at the center and some spindle fibroblastoid adherent cells at the periphery (elongated with tapering ends) at day 3 and day 4 (Fig. 6a, b). BM-MSCs samples showed adherent spindle-shaped fibroblasts-like cells, rapidly dividing cells at days 5‑7 following removal of non-adherent cells. Colonies of proliferating cells were seen radiating out of the explants after removing non-adherent cells at day 3 (Fig. 6c, d).

**Fig. 7:**
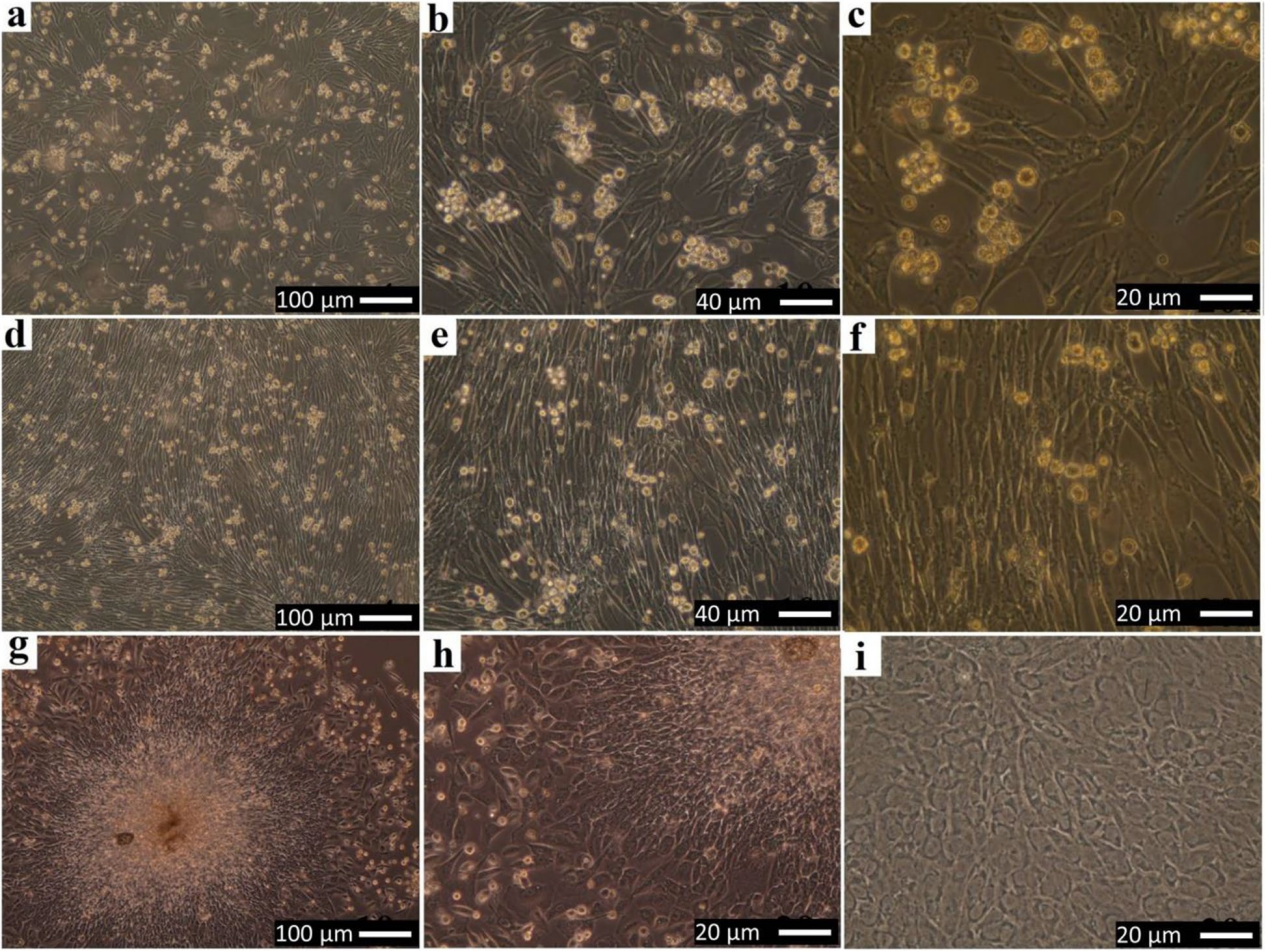
Inverted phase-contrast micrographs show the morphology of sub-cultured Rabbit BM-MSCs in the osteogenic medium in polystyrene culture plate as a negative control material (**a**-**c**) at day 3 and (**d**-**i**) at day 7, first passage through the first week of differentiation

The plastic adherence and morphology of isolated cells were compatible with undifferentiated MSCs. The adherent spindle-shaped fibroblasts-like cells proliferated and expanded rapidly and reached confluence (80‑90%) by days 5‑7 (Fig. 6e) at which time that the population was trypsinized and passaged. The confluence appeared as a fibroblastoid cell population around nodules in different fields of the flask and not the whole flask was a confluence. Nodular confluence was more important than flask confluence. The number of colonies varied from 3‑7 per flask T25 cm^2^. After subculture in 24 well plates (Fig. 6f), the morphology of some cells within the population grown in osteogenic medium (non-toxic control material) approximately changed in the first 48‑72 h following the addition of the osteogenic medium. Cuboidal cells protruding from the monolayer cell culture were observed. Rabbit BM-MSCs assumed a less elongated, polygonal, cuboidal appearance with central rounded nuclei or multipolar at day 3 (Fig. 7a‑c), and the cells showed coalescing cellular aggregates arranged in swirling sheets and bundles with interconnected multilayer foci showing central matrix-like substance like nodules at day 7 (Fig. 7d‑i).

### In vitro biocompatibility

**Fig. 8:**
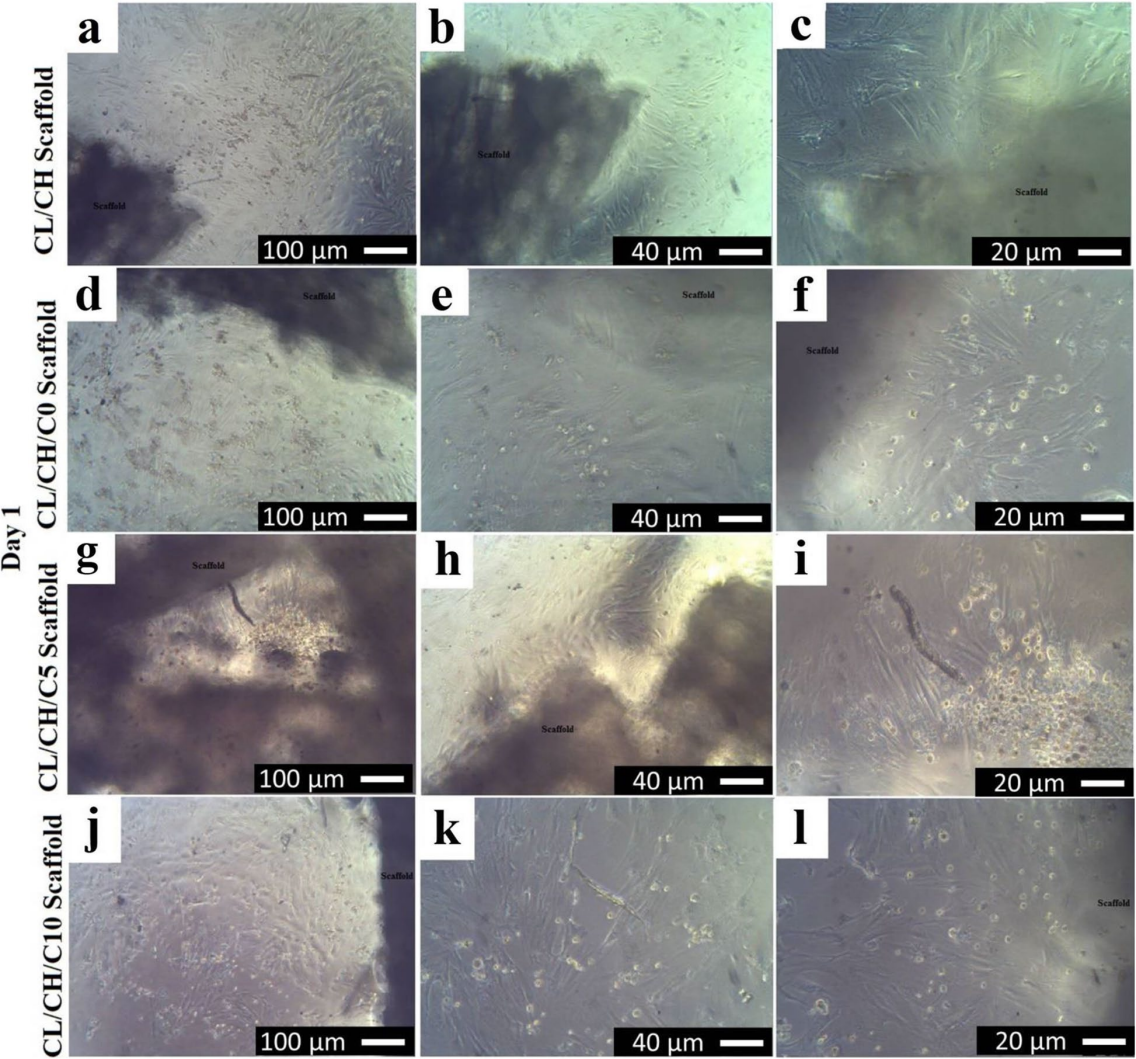
Inverted phase-contrast micrographs show the morphology of sub-cultured Rabbit BM-MSCs in osteogenic medium (direct contact) toxicity test of the four composite scaffolds (**a**-**c**) CL/CH, (**d**-**f**) CL/CH/C0, (**g**-**i**) CL/CH/C5, and (**j**-**l**) CL/CH/C10) respectively

**Fig. 9:**
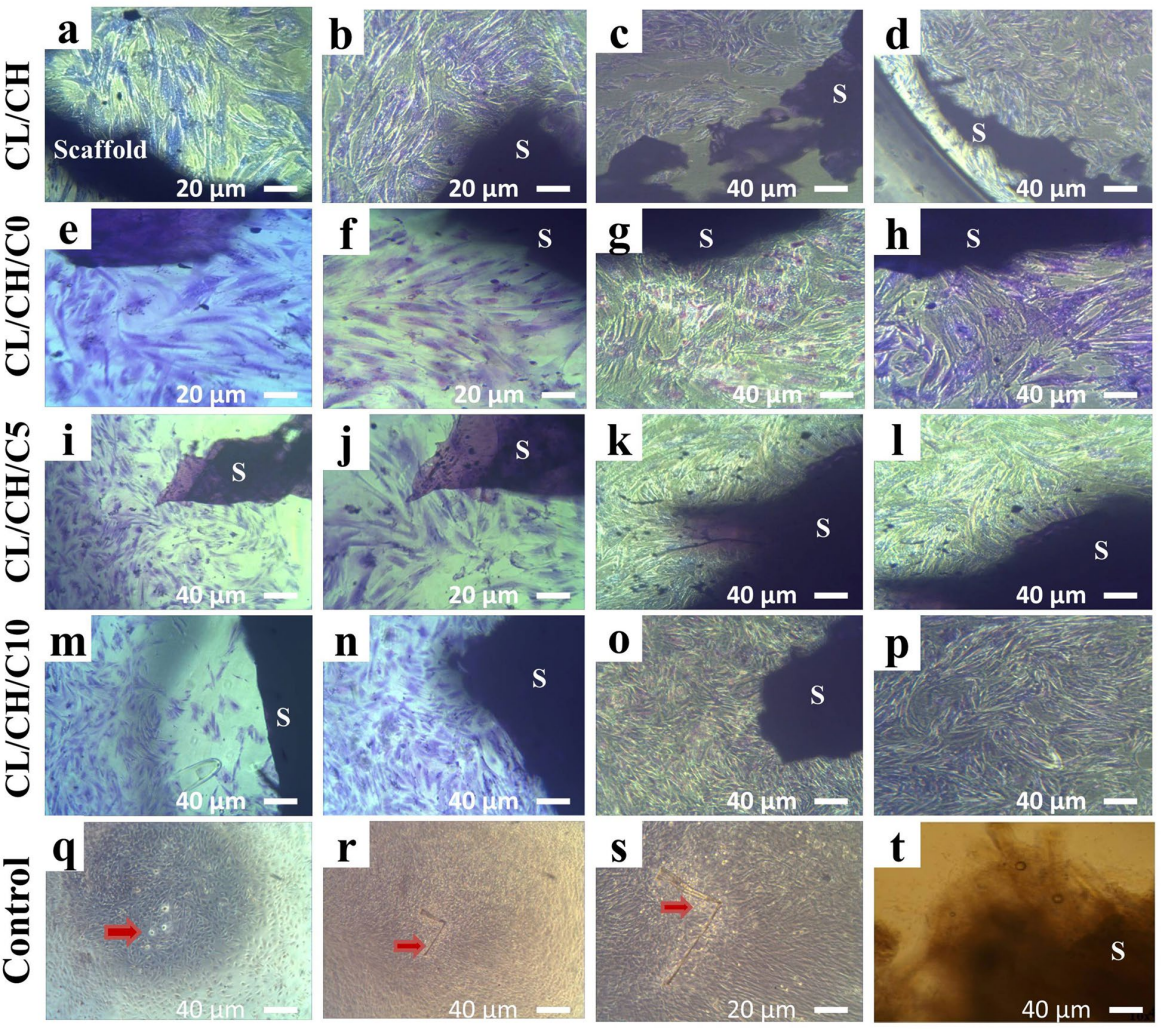
Inverted phase-contrast micrographs show the morphology of sub-cultured Rabbit BM-MSCs in osteogenic medium (direct contact toxicity test) of the four composite scaffolds by Giemsa stain at day 3 (**a**) CL/CH, (**e**-**f**) CL/CH/C0, (**i**, **j**) CL/ CH/C5, (**m**, **n**) CL/CH/C10 at day 7 (**b**-**d**) CL/ CH, (**g**, **h**) CL/CH/C0, (**k**, **l**) CL/CH/C5, and (**o**, **p**) C/ LCH/C10) of incubation the cells with the four composite scaffolds compared to (**q**-**s**) cells of negative control material, (**t**) plain unseeded scaffolds

**Fig. 10:**
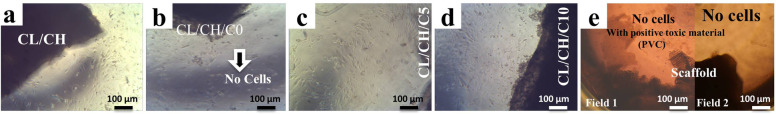
Inverted phase-contrast micrographs show the morphology of the first passage Rabbit BM-MSCs (direct contact toxicity test) at day 3 (**a**) CL/CH, (**b**) CL/CH/C0, (**c**) CL/CH/C5, and (**d**) CL/CH/C10 scaffolds supplemented with PVC mg/ml, (**e**) at day 7 100% (no cells were observed in well culture plates with PVC mg/ml

After the direct contact of the first passage of BM-MSCS in osteogenic medium with the four composite scaffolds for 1 day, the cells spread around the scaffold surfaces, the cell morphological changes, and increase in cell proliferation were observed obviously, especially around, in the vicinity and under the scaffold surfaces (Fig. 8 (a‑c) CL/CH, (d-f) CL/CH/C0, (g‑i) CL/CH/C5, and (j‑l) C/LCH/C10). Initially, many cells incubated with all scaffolds for 24 h were rounded in their shapes, and then the cells became spindle in morphology and increased in density with time. All the composite scaffolds showed proliferation of cells > 50%. Cells round in shape was in a state of synthesis of DNA (cell mitosis) which indicated that the cells had strong generation abilities. The number of spindles with fibroblast-like cells was increased more in density with the times especially around scaffold surfaces. From the Giemsa stain, it was clear that the cell proliferation was increased as the percentage of cerium in the glass incorporated into the scaffold also increased; all composite scaffolds showed proliferation of cells > 70%. A large number of cells proliferate, migrate, and spread into nearly every corner of the porous material at day 3 (Fig. 9 (a) CL/CH, (e‑f) CL/CH/C0, (i, j) CL/CH/C5, and (m, n) CL/CH/C10) and on day 7 the population of the cells increased manifestly (Fig. 9 (b‑d) CL/CH, (g, h) CL/CH/C0, (k, l) CL/CH/C5, and (o, p) CL/CH/C10). The morphology of the cells/scaffolds at day 3 and day 7 was similar to the cells of the non-toxic negative control material. While the cells of negative control material were concentrated in the center of good culture and began to form a dense culture with foci; central matrix-like substance with different shapes defined as nodules (Fig. 9q‑s), plain unseeded scaffolds were the same for all four composite scaffolds in culture medium (Fig. 9t). Another hand, PVC 20 mg/ml and PVC 100 mg/ml with four composite scaffolds showed inhibition of cell proliferation more than 80% at 24 h, cells death was observed (Fig. 10 (a) CL/CH, (b) CL/CH/C0, (c) CL/CH/C5, and (d) CL/CH/C10) and 100% at day 7 (no cells were observed in well culture plates and all wells became similar to plain unseeded scaffolds (Fig. 9t).

**Fig. 11:**
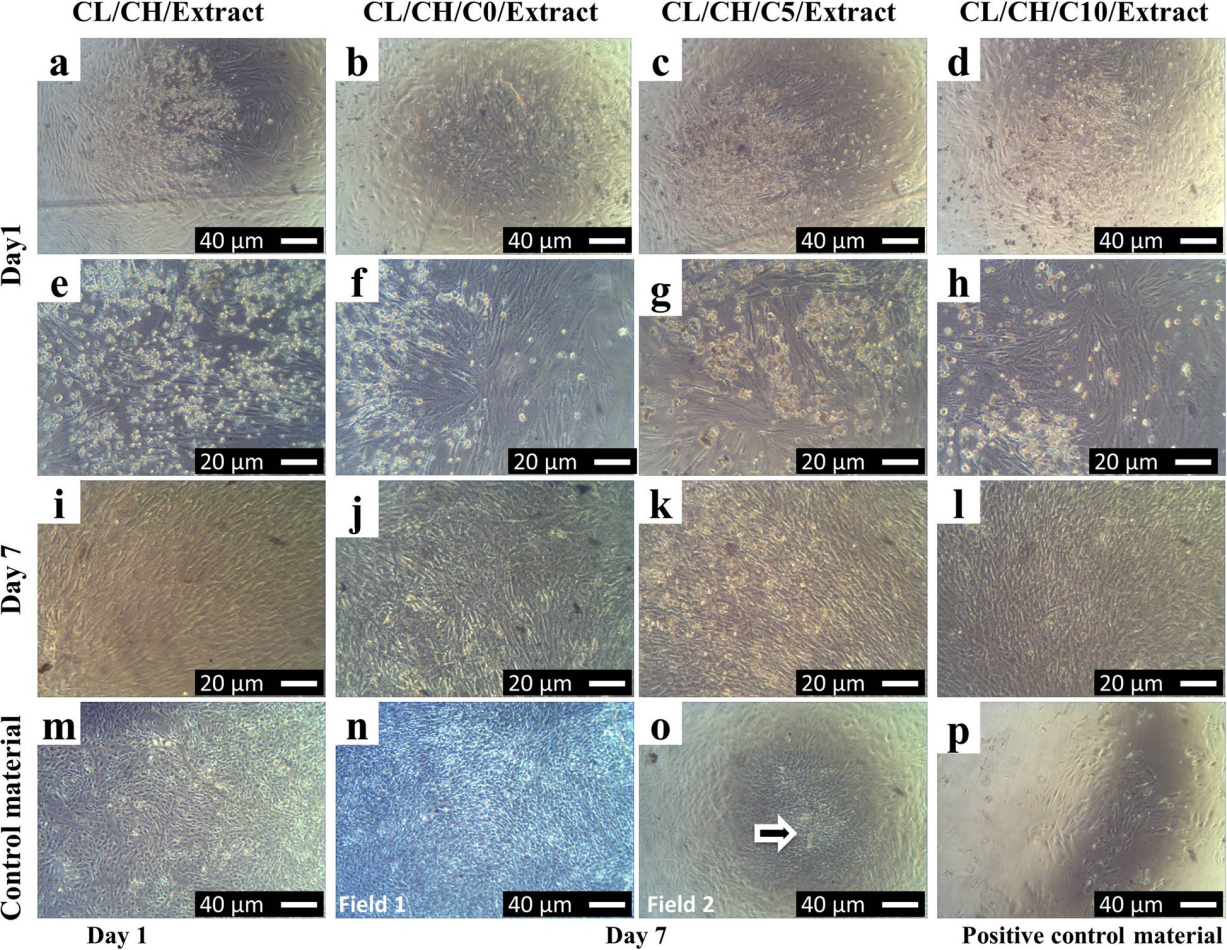
Inverted phase-contrast micrographs show the morphology of the first passage Rabbit BM-MSCs in osteogenic medium (indirect contact toxicity test) of the four composite scaffolds extracts at day 1 after 24 h of incubation of cells with extracts (**a**, **e**) CL/CH, (**b**, **f**) CL/CH/C0, (**c**, **g**) CL/CH/C5, (**d**, **h**) CL/CH/C10 at 10× 20× respectively and (**i**‑**l**) at day 7; the morphology was the same for all extracts respectively, (**m**‑**o**) negative control at day 1 and day 7 and (**p**) PVC, after 24 h of culture with four composite extracts

On the other side, cells proliferated well in the extracts of the four composites scaffolds (Fig. 11 (a, e) CL/CH, (b, f) CL/CH/C0, (c, g) CL/CH/C5, and (d, h) CL/CH/C10) at magnification 10×, 20× respectively for all composite extracts. Although many rounded cells and few changes in cell morphology were observed after 24 h of seeding cells with scaffolds extracts in culture, with time, the cells settle and cell morphology became the same with four extracts at day 7 (Fig. 11i‑l) and was similar to the cells of the non-toxic negative control material (Figs. 9q‑s and 11m‑o) at the same days. Positive control, PVC 20 mg/ml and 100 mg/m, affected the viability and proliferation of cells with an inhibition value > 90 % (toxic). Most cells died and detached from the bottom of the culture plate and then floated and removed after the first change of media (Fig. 11p). On day 7, no cells were observed in culture wells with (positive control material) in all extracts as showed before in field 2 (Fig. 10e). These results showed no toxicity presented in the extracts of scaffolds; the positive control material (PVC) was severely toxic and caused a marked detachment and death in cell culture.

#### Cell proliferation assay

**Fig. 12:**
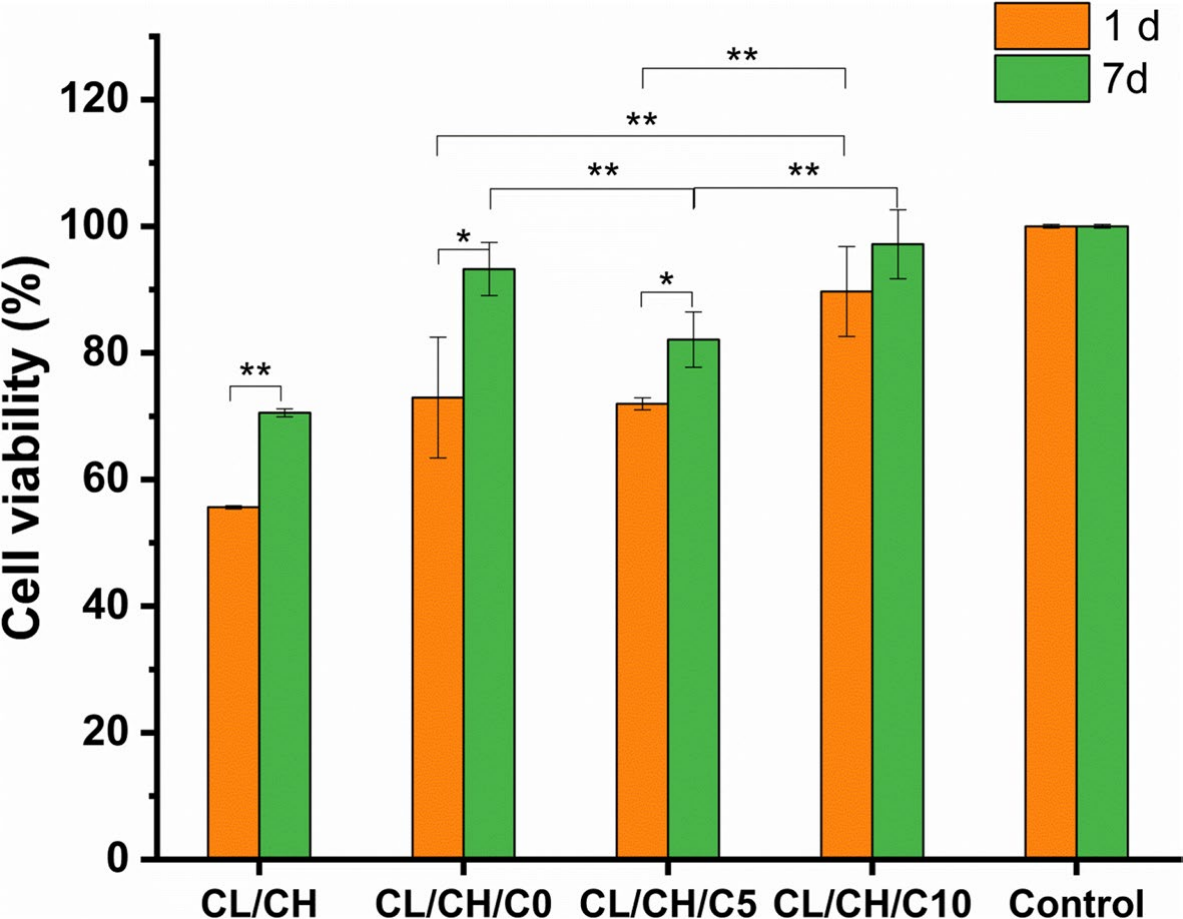
MTTassay of CL/CH/, CL/CH/C0, CL/CH/C5, and CL/CH/C10 composite scaffolds evaluated by proliferation of Rabbit BM-MSCs in osteogenic medium at day 1 and day 7. All composite scaffolds showed proliferation inhibition < 25% at day 7 reference to final cell number of control negative material (*P < 0.05 and **P <0.01)

MTT assay is an important method to evaluate the cytotoxicity of material where the scaffolds were slowly released components in the aqueous environment. Figure 12 shows the results of the MTT assay on day 1 and day 7, which reflected the number of viable cells (cell proliferation) of the scaffolds. The tissue culture plastic polystyrene with culture media with cells was used as a non-toxic, negative control material and PVC 20 mg/ml and 100 mg/ml as positive control material. The statistical analysis of cell proliferation using one-way ANOVA test showed that the difference of cell proliferation at day 1 and day 7 of culturing was significant (*p* < 0.05) for CL/CH, CL/CH/C0, and CL/CH/C5 scaffolds, while it was insignificant for CL/CH/C10. Moreover, the addition of nanobioactive glass particles (with/without Ce ion) to the scaffold polymer matrix increased the cell viability significantly (*p* < 0.05) either after 1 day or 7 days compared to the neat collagen/chitosan neat polymer scaffold. Meanwhile, at initial scaffolds cell culturing (1 day), the cell viability difference between CL/CH/C0 and CL/CH/C5 was insignificant, while it became significant (*p* < 0.01) when the CeO_2_ percentage increased to 10% (sample CL/CH/C10). Hence, the effect of Ce content to increase the cell viability of the scaffolds containing bioactive glass particles was significant when CeO_2_ content was 10 mole%, whereas the difference between these two scaffolds was insignificant after 7 days of cell culture. This can be explained by the consumption of Ce ions to interact with phosphate ions in the medium to form insoluble CePO_4_ crystals and thus decrease cell viability [41].

**Fig. 13:**
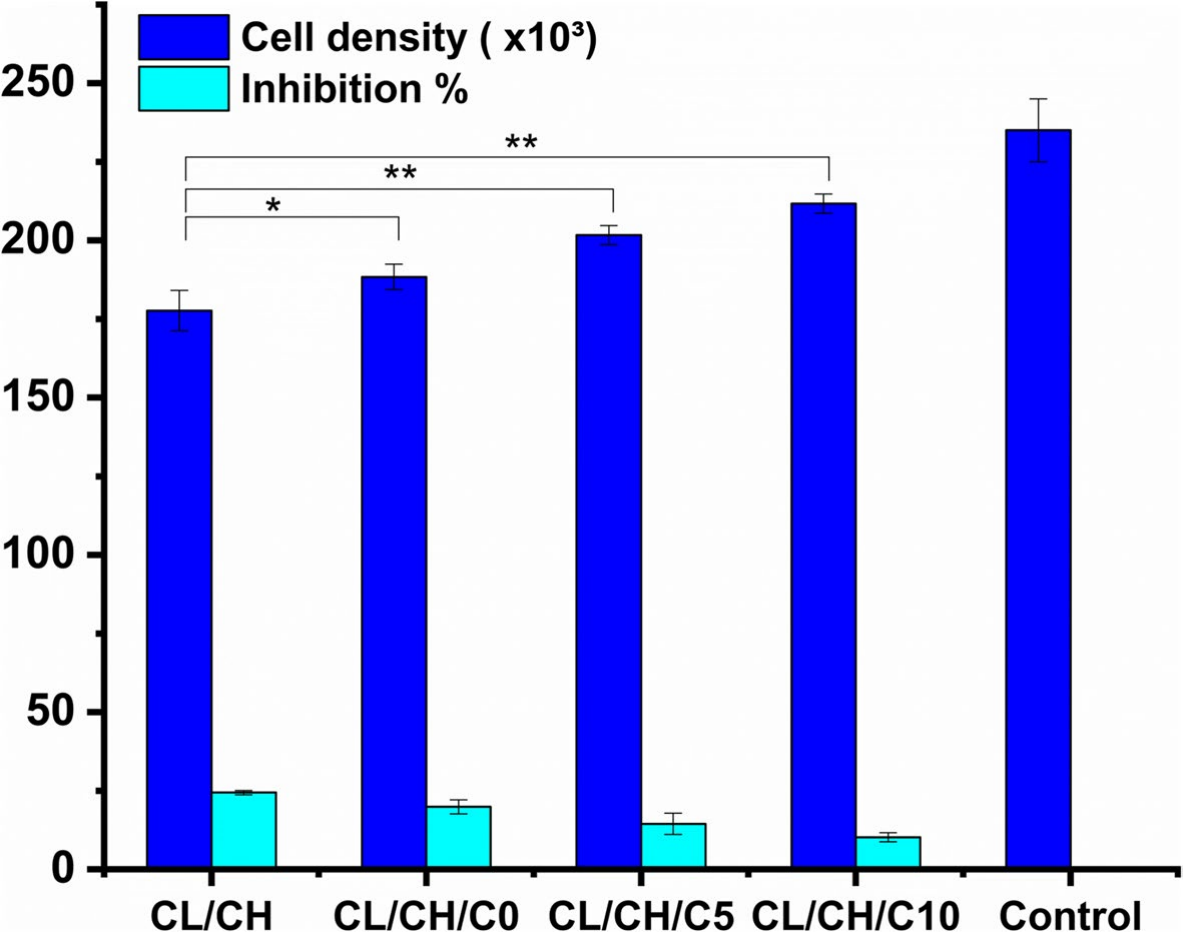
Toxicity effect of CL/CH, CL/CH/C0, CL/CH/C5, and CL/CH/C10 composite scaffold extracts evaluated by the proliferation of Rabbit BM-MSCs in the osteogenic medium after 7 days. All scaffolds extracts showed proliferation inhibition < 25% reference to final cell number of negative control material (*P < 0.05 and **P <0.005)

On the other hand, in the indirect contact test, few inhibitions of cell proliferation < 25% was detected for all prepared composite scaffold extracts by trypan blue and hemocytometer at day 7. This can be attributed to the dissolution of the scaffold materials producing components causing a relatively small change in the cell environment which might change cell viability (Fig. 13).

## Discussion

Recently, the cytotoxicity tests using cell lines are commonly used to estimate the biocompatibility of implanted biomaterials such as scaffolds, especially. Mouse fibroblast L929 and human osteoblast-like cell (HOB) are the most common cell line for bone regeneration purposes [71]. In this study, the cytotoxicity of the various composite scaffolds based on collagen and chitosan as natural polymer and nanobioactive glass doped with different ratios of CeO_2_ as a bioactive material was evaluated by using rabbit BM-MSCs directed to osteogenic lineage. The biocompatibility of the four composite scaffolds was evaluated in vitro by observing the behavior of the first passage of BM-MSCs cultured in osteogenic medium close contact around or in the vicinity of the four composite scaffolds and other with its extracts using phase-contrast microscopy and MTT assay. These two combined analyses give an overview for direct correlation between toxicity, cell death, reduced cell proliferation, and altered morphology [71, 72].

In the other words, polymer-ceramic composites are favorite candidates when aiming to replace bone tissue. Most natural polymers like collagen and chitosan proved to possess good biocompatibility and osteogenesis. Also, some ceramics, among which calcium phosphates (hydroxyapatite, the mineral phase of bone) and bioglass, have been shown positive effects on bone formation and cell fate in vivo and in vitro [73–80]. Nevertheless, there was no biocompatibility data of scaffold incorporated with NBG doped with different concentrations of CeO_2_.

The scaffold which caused cell death > 50% is considered toxic and unsafe to be used in biomedical applications [79]. Toxicity was tested by the death of 50% of cells (inhibition value > 50/IC50). In our study, at 24 h after direct contact with scaffolds, little change in the cell morphology. Most of the cells became rounded, especially, cells in the vicinity of scaffold surfaces, after that, the cells settled and became spindle and elongated. Little decrease in cell number, no cell death was observed. The inhibitions of cell proliferation and change of cell morphology occurred directly with the scaffolds supplemented with PVC 20 mg/ml and 100 mg/ml were > 90%. The inhibition was increased manifestly before reaching to day 7 until no attached cells were observed in the culture well (inhibition value 100%). The scaffolds under investigation did not show a negative effect on cell morphology and activity, and so, they were nontoxic for cells. Generally, the scaffold is considered cytocompatible when it does not cause changes in cell morphology and activity [80].

Moreover, the effect of scaffolds extracts on the morphology of BMMSCs in the osteogenic medium was also evaluated using a phase-contrast microscope; an extremely useful technique for observing specimens that were not been stained and were in their natural state. Obviously, the extracts of all composite scaffolds did not show a negative effect on the cell morphology, viability, and proliferation. Many rounded cells were observed with the four extracts were composed of scaffold materials resulting directly after 24 h of the culture of cells scaffolds extracts. Cells round in shape was in a state of synthesis of DNA (cell mitosis) [60], which indicated that the cells were having strong generation abilities as indicated on day 7 with quantities’ test, MTT assay for the four composite scaffolds (direct contact test) and proliferation assay (indirect contact test) using trypan blue and hemocytometer counting; rapid count generates accurate cell counts and viability results in seconds for the four scaffolds extracts. Assays revealed that none of the composite scaffolds and their extracts were negatively affected on the viability of the cells (inhibition value < 25%), and few deaths of the cells were observed. One possible explanation was weakness in a cellular environment (quality cells, culture media, and condition of incubation) in addition to the dissolution of scaffold materials result in cellular environment changes that reduce cell viability, all this must be taken into consideration that may affect the interpretation of scaffold toxicity if the calculation is based only in final cell count. Therefore, in this study, an inhibition percentage was determined by reference to the final cell number in control groups and compared to the four composite scaffolds and their extracts CL/CH, CL/CH/C0, CL/CH/C5, and CL/CH/C10 which supplemented with PVC 20 mg/ml and 100 mg/ml and PVC showed inhibition of cell proliferation more than 90%. This result was consistent with the result of the study, which reported previously [60], in which the authors stated that there was no cellular toxicity, impairment of cell adhesion to the plastic substrate (negative control), and decrease in cell viability found in the positive control, PVC was responsible for the cytotoxicity [60].

Testing of cell inhibition for composite scaffolds and its extracts showed that the inhibition was ordered from high to low as CL/CH>CL/CH/C0>CL/CH/C5>CL/CH/C10. Accordingly, osteoblasts proliferation was higher by incorporation of bioactive glass nanoparticles with polymer than the neat polymer, which indicated that the bioactive glass had stimulating effects to promote cell proliferation rate. This result was confirmed by several previous studies that used mainly natural polymers collagen and chitosan for bone regeneration and their conclusion for bone regeneration purposes was that collagen and chitosan polymers need bioactive material which can mimic the natural function of bone and improve unfavorable properties of natural polymer collagen and chitosan such as loss of osteoinductivity, low osteogenesis of collagen [9] and loss of osteoconductivity of chitosan which considers a very important property for bone healing in vivo after that [13]. Also, the addition of glass nanoparticles to the polymer was showed a considerable effect on the pore morphology and the pore size of the final scaffolds that characterized collagen/chitosan scaffold (sample CL/CH) with circular and relatively large pore size (50–300 μm), while the scaffolds contained bioactive glass fillers were characterized by elongated and a relatively small pore size. The biocompatibility of the scaffold was increased as the percentage of CeO_2_ bioactive glass nanoparticles increased, specifically, at 10 mole% of CeO_2_. The result is in agreement with previous observations on other Ce-containing biocomposite systems, wherein the bone-cell responses of the scaffold were enhanced by incorporating a suitable amount of Ce ions [81]. Seal and co-workers reported that Ce ions could increase the production of Collagen by human mesenchymal stem cells (HMSCs) cultured on porous bioactive glass scaffolds [81]. Porous bioactive glass scaffolds, which have been used clinically, can bind to the bone and act as a temporary guide and stimulus for bone growth in three dimensions.

Bone marrow-derived HMSCs line is a crucial cell type for bone regeneration in vivo because they differentiate into osteoprogenitor cells. It was reported that HMSCs are sensitive to toxic compounds derived from molecular oxygen. Ce ions can increase the proliferation of HMSCs by neutralizing oxidative stress [81]. Thus, embedding these ions into porous three-dimensional bioactive glass foam scaffolds could enhance osteoblastic differentiation of HMSCs and collagen formation. So, high Ce ion concentrations in samples exhibit cell viability higher than low concentration. These findings can be confirmed by the result of FTIR of the four composite scaffolds which indicated that the addition of CeO_2_ at expense of CaO was disrupted the silicate glass network and increased NBOs throughout the network [68]. The presence of a large number of NBOs increases the reactivity of the glass surface in the biological fluid and hence stimulates apatite formation with the surrounding tissues [69].

Hence, the biological effect of Ce ions in vitro and in vivo analyses contributes to its antioxidant activity. The effect of composite scaffolds containing CeO_2_ on the biological response might be ascribed either to the chemical nature of Ce ion or from the physical and morphological changes in roughness and stiffness brought to the composite scaffolds.

In the present study, with time through 1 week, the cell viability tests of the Ce-doped nanobioactive glass composite scaffolds and its extracts showed little inhibitions. This confirmed on high biocompatibility and also the early bioactivity of the composite scaffolds and their extracts was characterized by changing the BMMSCs morphology to osteoblast-like cells in a culture plate accompanied by the appearance of some early markers of osteoblast cells such as bone nodule formation while other markers are not detected in the present study. The bioactivity of these scaffolds and their extracts were discussed in detail in a complementary accepted study; the main target of it was the study of the bioactivity effect of these composite scaffolds toward osteogenic differentiation of normal rabbit’s osteoblast cells derived from bone marrow mesenchymal stem cells and studying also anticancer activity.

Accordingly, these findings showed that incorporation of Ce-doped nanobioactive glass in collagen/chitosan polymeric scaffolds increased proliferation of BMMSCs cultured under osteogenic condition for osteogenic lineage, hence, demonstrated a great potential for basic research and for future bone tissue engineering applications, especially when CeO_2_ was reported to exhibit positive effects (such as scavenging reactive oxygen species, ROS in other studies) [82] and so, the composite scaffolds were regarded as promising bioactive materials for biomedical applications.

## Conclusions

Nanobioactive glass supported the attachment and proliferation of BM-MSCs, and provided an appropriate environment for cell proliferation. The incorporation of CeO_2_ in the glass composition which used as a bioactive filler of collagen/chitosan scaffolds increased cell viability and proliferation. The composite scaffold with the highest content of CeO_2_ content (CL/CH/C10) showed the least toxic effect to BM-MSCs directed to osteogenic lineage. Ce ions were enhanced the early osteogenic proliferation that can be used for studying the bone mineralization process after that. Thus, these findings showed that the prepared hybrid Ce-doped glass/collagen/chitosan bioactive scaffolds were biocompatible and hold great potential for basic research and bone tissue engineering applications.

## Supplementary Information

**Additional file 1.**

## Data Availability

Not applicable for this articles.
