# Supplementary material for: Effect of Ce-doped bioactive glass/collagen/chitosan nanocomposite scaffolds on the cell morphology and proliferation of rabbit’s bone marrow mesenchymal stem cells-derived osteogenic cells
Source: J Genet Eng Biotechnol. 2022 Feb 21;20:33. doi: 10.1186/s43141-022-00302-x (PMC8864049; doi:10.1186/s43141-022-00302-x)
Supplement: Supplementary file 1 — Additional file 1. [file 43141_2022_302_MOESM1_ESM.pdf]

The ARRIVE Essential 10: Compliance Questionnaire

Use this questionnaire to evaluate how well a manuscript complies with the ARRIVE Essential 10. It can be applied to any manuscript describing comparative experiments in living animals, by assessors such as journal staff, editors, or peer reviewers.

Item	Question(s)	Answers
1 Study Design	Are all experimental and control groups clearly identified?	☒ Yes, for at least one experiment<br>☐ No Methods, paragraph 3
	Is the experimental unit (e.g. an animal, litter or cage of animals) clearly identified?	☒ Yes, for at least one experiment<br>☐ No Methods, paragraph 3
2 Sample Size	Is the exact number of experimental units in each group at the start of the study provided (e.g. in the format '*n*=')?	☒ Yes, for at least one experiment<br>☐ No Methods, paragraph 5
	Is the method by which the sample size was chosen explained?	☒ Yes, for at least one experiment<br>☐ No Methods, paragraph 5
3 Inclusion & Exclusion Criteria	Are the criteria used for including and excluding animals, experimental units, or data points provided?	☒ Yes, for at least one experiment<br>☐ No N/A
	Are any exclusions of animals, experimental units, or data points reported, or is there a statement indicating that there were no exclusions?	☒ Yes, for at least one analysis<br>☐ No N/A
4 Randomisation	Is the method by which experimental units were allocated to control and treatment groups described?	☒ Yes, for at least one experiment<br>☐ No Methods, paragraph 3
5 Blinding	Is it clear whether researchers were aware of, or blinded to, the group allocation at any stage of the experiment or data analysis?	☒ Yes, for at least one experiment<br>☐ No N/A
6 Outcome Measures	For all experimental outcomes presented, are details provided of exactly what parameter was measured?	☒ Yes, for at least one experiment<br>☐ No Results, paragraphs 1-6
7 Statistical Methods	Is the statistical approach used to analyse each outcome detailed?	☒ Yes, for at least one analysis<br>☐ No Results, paragraphs 5, 6
	Is there a description of any methods used to assess whether data met statistical assumptions?	☐ Yes, for at least one analysis<br>☐ No<br>☒ Not applicable N/A
8 Experimental Animals	Are all species of animal used specified?	☒ Yes, for at least one experiment<br>☐ No Methods, paragraph 3
	Is the sex of the animals specified?	☒ Yes, for at least one experiment<br>☐ No Methods, paragraph 3<br>☐ Not applicable to species
	Is at least one of age, weight or developmental stage of the animals specified?	☒ Yes, for at least one experiment<br>☐ No Methods, paragraph 3
9 Experimental Procedures	Are both the timing and frequency with which procedures took place specified?	☒ Yes, for at least one experiment<br>☐ No Methods, paragraph 3
	Are details of acclimatisation periods to experimental locations provided?	☒ Yes, for at least one experiment<br>☐ No N/A
10 Results	Are descriptive statistics for each experimental group provided, with a measure of variability (e.g. mean and SD, or median and range)?	☒ Yes, for at least one experiment<br>☐ No Results, paragraphs 5, 6<br>☐ Not applicable to the type of data collected
	Is the effect size and confidence interval provided?	☐ Yes, for at least one experiment<br>☐ No N/A<br>☒ Not applicable to the type of analysis used

Notes on questionnaire design

The ARRIVE guidelines are a useful resource for authors preparing manuscripts describing animal research, and also provide a framework to evaluate the transparency of those manuscripts. To assess reporting quality, numerous studies have in the past sought to operationalise reporting guidelines (including ARRIVE). Typically, this involves scoring a manuscript's degree of compliance with guideline items in a binary fashion (e.g. an item is either not reported or reported) [1-3], a graded fashion (e.g. not, partially, or completely reported) [4,5], or a combination of the two [6].

This questionnaire has been designed to be as concise and user-friendly as possible. The number of questions used to assess a manuscript's compliance has been kept to a minimum, and in most cases each question is designed to be answered in a binary fashion. Compliance with some Essential 10 sub-items is inherently impossible to judge in this way, instead requiring a subjective judgement on the level of detail provided. For this reason, not all sub-items are represented by a question in this questionnaire.

To facilitate binary answers, it has been necessary to identify the minimum information in a manuscript sufficient to comply with each question. The strengths of this approach include the relatively short length of the questionnaire (and the correspondingly low time burden of using it), and the avoidance of ambiguity that would arise from a graded answering system, in which an intermediate score (e.g. 'partially/insufficiently reported') could denote a number of distinct deficiencies in compliance with an item (e.g. either only part of the item was complied with, or only the reporting of some experiments in the manuscript complied with the item.)

Limitations of this approach centre on the necessity to identify the minimum information sufficient to comply with each question. In some cases, this has resulted in questions that require a guideline sub-item's criteria to have been fulfilled in the reporting of only one experiment in a manuscript. As a result, not all experiments in a manuscript may be described in a way that fulfils that criterion, despite the manuscript being considered to comply with the guidelines overall.

References

1. Hair *et al* (2020). *Res Integ Peer Rev*. doi: 10.1186/s41073-019-0069-3
2. Tihanyi *et al* (2019). *J Surg Res*. doi: 10.1016/j.jss.2018.10.038
3. Zhao *et al* (2020). *BMC Vet Res*. doi: 10.1186/s12917-020-02664-1
4. Han *et al* (2017). *Plos One*. doi: 10.1371/journal.pone.0183591
5. Chatzimanouil *et al* (2019). *J Am Soc Nephrol*. doi: 10.1681/ASN.2018050515
6. Leung *et al* (2018). *Plos One*. doi: 10.1371/journal.pone.0197882
